# Somatostatin interneuron inhibition as a strategy to restore excitation‐inhibition balance in Alzheimer's disease

**DOI:** 10.1002/alz70855_106574

**Published:** 2025-12-24

**Authors:** Moustafa Algamal, Sarena Abdallah, Wadzanai Ndambakuwa, Ksenia V. Kastanenka, Brian J. Bacskai

**Affiliations:** ^1^ Harvard Medical School, Boston, MA, USA; ^2^ Massachusetts General Hospital, Charlestown, MA, USA

## Abstract

**Background:**

Alzheimer's disease (AD) is characterized by the accumulation of extracellular amyloid plaques, leading to aberrant neuronal activity and an imbalance between excitatory and inhibitory activity. This disruption contributes to network dysfunction and cognitive impairment in AD patients. Our previous work has demonstrated that excitatory neuron activity is reduced in APP/PS1 mice, while somatostatin (SOM) inhibitory interneuron activity is increased near amyloid plaques under isoflurane anesthesia. Given this imbalance, we hypothesized that inhibiting SOM interneurons could restore excitatory neuron activity and improve memory deficits in AD mouse models. To test this, we employed both acute and chronic chemogenetic approaches to manipulate SOM interneuron activity.

**Methods:**

We used in vivo calcium imaging in APP and wild‐type (WT) mice to evaluate excitatory neurons after acute chemogenetic inhibition of SOM interneurons. For chronic inhibition, we intravenously delivered AAVPHP.eB encoding chemogenetic receptors to achieve non‐invasive, brain‐wide inhibition of SOM interneurons in APP/PS1 and WT mice. We then administered C21 subcutaneously for 16 days, followed by behavioral assessments. Behavioral tests evaluated locomotion and memory consolidation.

**Results:**

Calcium imaging confirmed that the excitation‐inhibition (E/I) imbalance persists in awake APP mice, demonstrating increased SOM interneuron activity and decreased excitatory neuron activity, mirroring our findings under anesthesia.

Acute chemogenetic inhibition of SOM interneurons successfully enhanced excitatory neuron activity, supporting the hypothesis that reducing SOM interneuron activity can restore E/I balance in APP/PS1 mice. However, chronic inhibition of SOM interneurons failed to restore behavioral deficits in APP/PS1 mice, as no significant differences were observed in locomotor activity, working memory, or fear memory acquisition and recall in APP/PS1 or WT mice.

**Conclusion:**

While acute inhibition of SOM interneurons can restore excitatory neuron activity, brain‐wide chronic inhibition does not effectively improve memory function in APP/PS1 mice under the current experimental conditions.

